# Interlimb Asymmetries Identified Using the Rate of Torque Development in Ballistic Contraction Targeting Submaximal Torques

**DOI:** 10.3389/fphys.2018.01701

**Published:** 2018-11-28

**Authors:** Gennaro Boccia, Paolo Riccardo Brustio, Giampiero Buttacchio, Marzia Calabrese, Marco Bruzzone, Roberto Casale, Alberto Rainoldi

**Affiliations:** ^1^NeuroMuscularFunction Research Group, School of Exercise and Sport Sciences, Department of Medical Sciences, University of Turin, Turin, Italy; ^2^Habilita Care & Research Rehabilitation Hospital, Bergamo, Italy; ^3^Medical Staff, Atalanta BC, Bergamo, Italy

**Keywords:** risk factor, imbalance, soccer, hamstrings, quadriceps

## Abstract

Evaluating the rate of torque development (RTD) in isometric ballistic contraction targeting submaximal torques is usually overlooked in the literature. In a series of isometric ballistic contractions targeting a range of submaximal torque values, there is a linear relationship between the peak torque and the peak RTD obtained in each contraction. RTD scaling factor (RTD-SF) represents the slope of this relationship. In this study, we investigated the prevalence of interlimb asymmetry in the RTD-SF and in the RTD calculated across submaximal torques. Furthermore, we compared these asymmetry indices with those calculated adopting more classical approaches, such as ballistic contraction targeting maximal torque and isokinetic concentric conditions. Quadriceps and hamstrings strength was evaluated in both limbs of elite under 17 and under 19 soccer players (20 males, 17 ± 1 years). Participants performed three concentric isokinetic contractions at 240°/s and a series of isometric ballistic contractions targeting from 20 to 100% of maximal isometric torque. The interlimb difference was calculated for each parameter and players presenting an interlimb difference >15% were identified. A total of 40% (for quadriceps) and 60% (for hamstring) of players showed an interlimb asymmetry in isometric RTD for at least 50% of submaximal torque range. The RTD-SF was able to identify more players with asymmetry than the classical isokinetic tests. However, isokinetic and isometric indices of asymmetry were in general poorly or not correlated with each other. Most players presented an interlimb asymmetry in RTD for a wide part of the torque range and the adopted protocol was able to highlight important interindividual differences. Furthermore, players showed a large prevalence of RTD-SF asymmetry in both quadriceps and hamstrings. It is still to be determined if these asymmetries are functionally relevant. Nevertheless, the adopted protocol provided meaningful information for identifying interlimb asymmetries that could not be gathered when adopting the classical method of ballistic contractions targeting only maximal torques.

## Introduction

The prevalence of interlimb asymmetries, also referred to as bilateral muscle strength asymmetry, has been reported in numerous studies across a wide range of sports. A relative strength difference greater than 10 or 15% between limbs has been adopted as criteria of interlimb asymmetry (Kannus, 1994; Impellizzeri et al., 2007; Jones and Bampouras, 2010). For example, using a cut-off of 15% as asymmetry criterion between lower limbs, Fousekis et al. (2010b) found that professional soccer players presented quadriceps or hamstring strength asymmetries (65 and 45%, respectively). Similarly, up to 56% subjects among young elite and professional soccer players presented interlimb asymmetry in quadriceps and hamstrings (Lehance et al., 2009). Other studies (Zakas, 2006; Ruas et al., 2015) did not find muscle strength asymmetry in lower limbs. Probably, the discrepancies found in the literature might be due to variability in testing protocol and evaluated cohorts as well as the difference in training age (Lehance et al., 2009; Fousekis et al., 2010a) and playing position (Ruas et al., 2015; Sliwowski et al., 2017).

Interlimb asymmetry evaluation has been used to assess injury risk in soccer players (van Dyk et al., 2018). Indeed, interlimb asymmetry has been considered a risk factor for lower limb injuries (Croisier et al., 2008; Fousekis et al., 2011; Green et al., 2018). For instance, Croisier et al. (2008) found that rate of hamstring strain injury in soccer players with muscle strength asymmetries and imbalance in quadriceps and hamstrings were nearly five times higher in comparison with players with muscular strength symmetry. Soccer players without previous injuries, but with an eccentric isokinetic strength asymmetry >15%, and a leg length asymmetry (i.e., >1.8 cm) had a higher likelihood of hamstring strain injury than those with lower or no asymmetries, but with a hamstring strain history (Fousekis et al., 2011). Nevertheless, a recent meta-analysis (Green et al., 2018) showed that interlimb asymmetry *per se*, has a limited predictive value to detect future hamstring muscle injuries. In addition, the assessment of interlimb asymmetry has been widely used to monitor the progress of rehabilitation process (Zwolski et al., 2016; Faltstrom et al., 2017). Indeed, it can be used to quantify the functional recovery after an injury/surgery (Impellizzeri et al., 2007). An interlimb asymmetry below the cut-off value 10% has been proposed as a safe criterion for returning to sport (Bishop et al., 2018). However, many professional soccer players still have a residual isokinetic strength deficit at the return to play (Tol et al., 2014). Finally, the role of interlimb strength asymmetry on soccer performance is less clear (for review see Bishop et al., 2018; Maloney, 2018). For example, while some studies showed that a greater interlimb difference in quadriceps strength was associated with faster sprint time and change of direction (e.g., Lockie et al., 2012), other studies showed the opposite (e.g., Coratella et al., 2018).

Generally, isokinetic dynamometry is a common method to assess quadriceps and hamstrings strength and to identify asymmetries (Croisier et al., 2007; Maffiuletti et al., 2007; de Carvalho Froufe Andrade et al., 2013). Recently the assessment of rate of force development or rate of torque development (RTD) has been adopted to evaluate the rapid strength characteristics. RTD assessment allows to evaluate the ability to exert force in a limited amount of time, which are typically required in soccer actions. RTD reflects the ability to quickly increase muscle force after the onset of an explosive voluntary contraction (Maffiuletti et al., 2016; Rodriguez-Rosell et al., 2017). RTD has been recently adopted to assess rapid strength deficits after anterior cruciate reconstruction in both injured (Knezevic et al., 2014) and contralateral limb (Mirkov et al., 2017). Thus, RTD has been proposed to be an adjunctive outcome measure for return-to-sport decisions (Angelozzi et al., 2012).

Rate of torque development has been usually assessed in ballistic contractions requiring participants to reach high level of torque, usually higher than 80% of maximal torque, while producing torque as quickly as possible (Folland et al., 2014). Throughout the manuscript we will consider the RTD measured following the above-mentioned procedure as maximal RTD (RTD_max_). Similarly to the assessment of RTD_max_, isokinetic strength assessment requires the subject to produce the maximal torque as quickly as possible. Although RTD_max_ and maximal isokinetic torque might convey important information, it may not reflect the demands of all technical conditions. Indeed producing the maximal torque as quickly as possible may be the target of explosive movements like change of direction or the vertical jump (McLellan et al., 2011), but this is not the case for many other technical movements. Indeed, soccer players often try to produce submaximal torque as quickly as possible, especially in situations that require precision such as passing, dribbling, and scoring (Thorlund et al., 2009). For this reason, while a deficit in producing ballistic contraction of maximal amplitude may be relevant for some technical movements, an impairment in capacity to quickly produce ballistic contractions of submaximal amplitude may be more relevant for some specific soccer skills. For the above-mentioned reasons, we suggest that latter ability has higher ecological validity than the RTD_max_. In this context, the protocol usually adopted to calculate the so-called RTD scaling factor (RTD-SF) provides an appealing approach (Bellumori et al., 2011, 2013; Casartelli et al., 2014; Djordjevic and Uygur, 2017; Boccia et al., 2018). The protocol consists of a series of ballistic (so called pulses) contractions performed with different submaximal amplitudes (namely from 20 to 100% of maximal isometric torque) (Freund and Budingen, 1978; Wierzbicka et al., 1991; Klass et al., 2008). In each ballistic contraction the individuals are asked to reach a submaximal torque as fast as possible and then to relax their muscles quickly. The RTD produced in each contraction thus provides a measure of the ability to quickly produce torque of submaximal amplitude (Bellumori et al., 2011, 2013; Casartelli et al., 2014; Djordjevic and Uygur, 2017; Boccia et al., 2018).

In the above-mentioned protocol, the RTD-SF specifically consists of the slope of linear relationship between the peak torque and the peak RTD obtained in each contraction (Bellumori et al., 2011, 2013; Casartelli et al., 2014; Djordjevic and Uygur, 2017). The studies available in the literature suggest that RTD-SF measurement informs about the important features of movement initiation and quickness of force production (Wierzbicka et al., 1991; Bellumori et al., 2011). However, the physiological mechanisms underpinning the RTD-SF are far from being elucidated. The few intervention studies demonstrated that RTD-SF improved with training on explosiveness and its improvement was related to an increase in motor units discharge rate in the early part of muscle contractions (Van Cutsem et al., 1998; Bellumori et al., 2017). Unlike RTD_max_, RTD-SF is independent of muscle strength (Bellumori et al., 2011; Boccia et al., 2018), which facilitates comparisons among different population and muscle groups.

We aimed to provide more data about the potential usefulness of assessing the RTD when targeting submaximal torques to identify interlimb asymmetry. Thus, the aim of this study was threefold:

(1) to determine the prevalence of interlimb asymmetry in the RTD in ballistic contractions of submaximal amplitudes;
(2) to determine the prevalence of interlimb asymmetry in RTD-SF;
(3) to compare the RTD-SF with other measurements usually adopted to identify asymmetry, namely maximal isometric torque, RTD_max_, and concentric isokinetic test. Specifically, we compared the prevalence of asymmetry adopting these indices and we calculated the correlations between them.

## Materials and Methods

### Participants

Twenty-two elite young soccer players (age 17 ± 1 years, body mass 72 ± 9 kg, height 1.82 ± 0.08 m) were recruited for this study. The participants joined under 17 and under 19 teams of a Serie A club in Italy. All the participants were healthy, without cardiac or pulmonary diseases, as certified by the club’s medical staff. Players with knee, ankle, or hip injury in the previous 6 months were excluded from the present investigation. Therefore, two out of 22 players were excluded from the present investigation. All participants provided their written informed consent before participation in the experiments. The study was approved by the local Ethical Committee and performed in accordance with the Declaration of Helsinki.

### Data Acquisition

**FIGURE 1:**
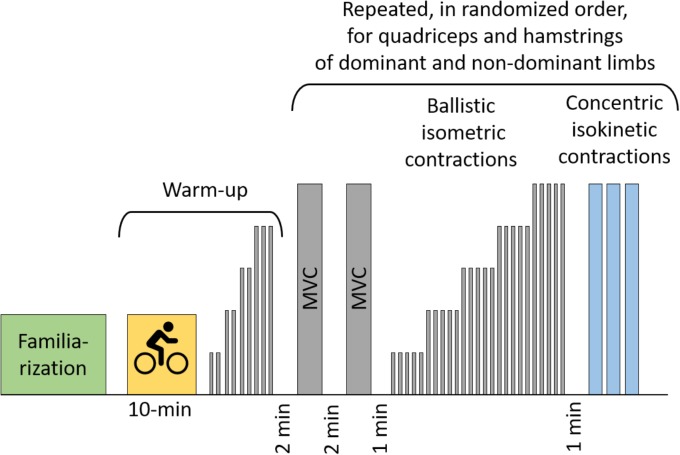
Schematic representations of the testing procedure. In gray are reported the isometric contractions. MVC, maximal voluntary contraction.

The present investigation was performed in pre-season. The strength of the quadriceps and hamstrings was measured by an isokinetic dynamometer (BIODEX System 3, Biodex medical system, NY, United States) in isometric and isokinetic conditions. The device was calibrated and the gravity correction executed according to the manufacturer’s procedures. The participants were seated with their trunk reclined at 85° and then secured by seatbelts. Thigh and tibia were secured using straps and the center of rotation of the dynamometer was aligned with the knee. The limb order was randomized, while quadriceps was always tested before hamstrings. Throughout the measurement session, visual feedback of the force output was provided as a signal displayed real time on a computer screen. A schematic representation of the protocol is reported in Figure 1.

### Procedure

**FIGURE 2:**
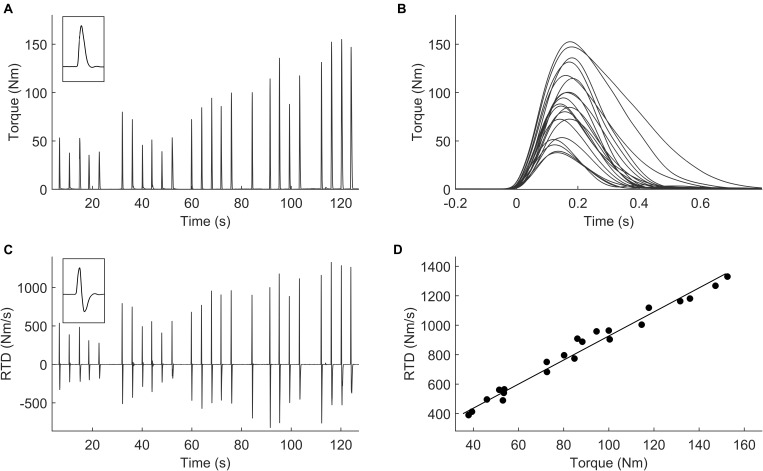
Representative example of a set of rapid force contraction (pulses) performed across a range of submaximal amplitudes. **(A)** Torque signals where are visible 5 or 6 pulses for each torque level; **(B)** RTD signals (first derivative of force); **(C)** superimposed torque pulses signals; **(D)** each point represents the peak RTD (*y*-axis) and the peak torque (*x*-axis) achieved in each pulse.

The warm up consisted of 10 min of cycling at 80 W and of 10 sub-maximal isometric contractions for quadriceps and hamstrings. Then the participants performed two maximal voluntary isometric contractions (MVC) separated by 2 min of rest. One minute after the last MVC, participants were requested to produce a series of isometric ballistic contractions across a full range of amplitudes (Figure 2). In each ballistic contraction the individuals were asked to roughly reach the given submaximal torque as quickly as possible and then to relax instantly (see Figures 2A,B) (Freund and Budingen, 1978; Wierzbicka et al., 1991; Klass et al., 2008; Bellumori et al., 2011). Participants performed four to six contractions at five different amplitudes presented in an ascending order (20, 40, 60, 80, and 100% MVC torque, see Figures 2A,B). The rest interval between contractions was 4 s.

During the execution of this protocol, the emphasis was on the quickness of the contraction rather than on the accurateness. Therefore, participants were explicitly instructed not to accurately target the given submaximal torque because accurateness slows the rate of torque production (Gordon and Ghez, 1987). Instead, they were asked to produce quick contractions with peak torques reaching approximately a 10% range around the given torque target. As previously reported (Bellumori et al., 2011, 2013; Casartelli et al., 2014; Boccia et al., 2018), in the familiarization session, participants practiced until they felt comfortable with the task and could perform discrete ballistic contractions as instructed.

One minute after the last isometric ballistic contraction (Figure 1), participants performed three consecutive maximal contractions in concentric modality at 240°/s. The range of movement in isokinetic contraction was from 80 to 10° (for quadriceps) and from 10 to 80° (for hamstrings), considering 0° = full knee-extension.

### Signal Analysis

The torque signal were sampled at 2048 Hz, converted to digital data with a 12-bit A/D converter (EMG-USB2+, OT Bioelettronica, Turin, Italy), and filtered by using a low-pass filter a with a cut off frequency of 50-Hz. Torque signals were analyzed by custom-written software in MATLAB R2015a (MathWorks, Natick, MA, United States). The MVC torque and the peak torque in concentric contraction (CONC) were calculated as the maximum of the torque signal recorded across the two or three attempts for each test.

To calculate the RTD in the ballistic contractions, the torque signal was firstly pre-processed using an overlapping moving window of 0.1 s (Bellumori et al., 2011). If a countermovement, i.e., a visible drop in torque, was performed before the torque onset, the contraction was discarded from the analysis. Then, the first derivative of the torque signal was computed to obtain the RTD signal (Nm/s, see Figure 2C). For each contraction, peak torque and peak RTD (which is local maximum of the RTD signal) were calculated. The RTD_max_ was considered as the RTD recorded during the contraction that presented the highest RTD.

### Statistical Analysis

#### Interlimb Asymmetry

Statistical analysis was performed by custom-written software in MATLAB R2015a (MathWorks, Natick, MA, United States). The interlimb asymmetry for each parameter was calculated according to the formula proposed by Impellizzeri et al. (2007): asymmetry (%) = (stronger limb-weaker limb)/stronger limb × 100. Differences between stronger and weaker limb were reported in absolute and percent values, the precision of estimates for absolute values was indicated with 90% confidence intervals. Data are presented as mean ± standard deviation (SD). Threshold for statistical significance was set at *p* < 0.05.

#### Linear Regression Between Torque and RTD

The linear regression between the peak torque and peak RTD obtained during the ballistic contractions was calculated (Figure 2D). Outliers were removed using the Cook distance methodology (Cook, 2000). The slope of the linear regression represents the RTD-SF and is usually considered as the main outcome for this protocol (Bellumori et al., 2011; Casartelli et al., 2014; Djordjevic and Uygur, 2017).

In 14 occasions out of 40 (20 participants for two muscles, i.e., quadriceps and hamstrings), the relationship between peak torque and peak RTD was not linear for the whole contraction range. Rather, it was linear from 0 to about 70–90% of the maximal torque and then showed a logarithmic behavior from about 70–90% to the maximum. In these occasions, biphasic regression explained more variation than a linear regression between torque and RTD (Linden, 2015). The breakpoint for this interrupted regression was calculated (Linden, 2015) and the coefficients for the first part of linear regression, i.e., up to 70–90% of maximal torque, was reported.

##### RTD in submaximal contractions

To answer the first experimental question of the study we compared the peak RTD produced at the same absolute torque level between limbs. Since it is virtually impossible to have two ballistic contractions with the same amplitude in right and left limb, we decided to evaluate the linear regression between RTD and peak torque at predetermined torque levels across the whole available range of torques. To do this, the regression line of the RTD-SF was evaluated from 1 Nm, with 1 Nm intervals, to the highest available peak torque. The range of torque values that presented an RTD interlimb asymmetry greater than 15% was calculated and reported as a percentage of the whole torque range.

##### RTD-SF

To answer the second experimental question, the percentages of players who did not achieve the five different cut-off criteria of 5, 10, 15, 20, and 25% of interlimb asymmetry for RTD-SF were identified.

##### Comparison between indices of asymmetry

To answer the third experimental question, we compared the prevalence of interlimb asymmetry (using the cut-off criteria of 5, 10, 15, 20, and 25%) between RTD-SF and the other asymmetry indices, namely RTD_max_, MVC torque, and CONC. Furthermore, Pearson correlation coefficient *r* was used to compare RTD-SF with the interlimb asymmetry indices.

## Results

### RTD in Submaximal Contractions

**FIGURE 3:**
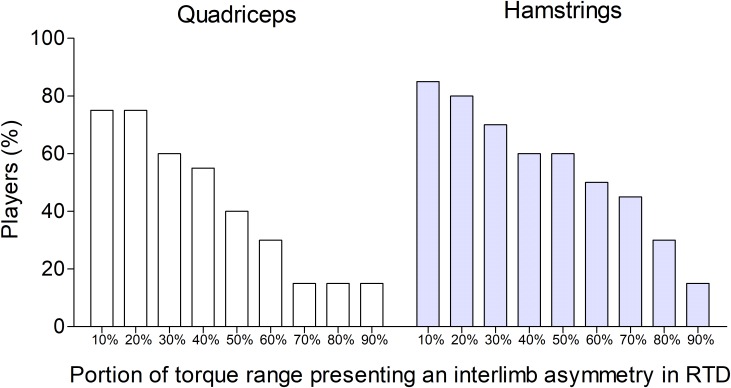
Percentage of players (*y*-axis) showing an interlimb asymmetry in rate of torque development (RTD) across the submaximal torque levels. The *x*-axis represents the percentages of torque range (from 10 to 90%) in which the interlimb asymmetry in RTD was larger than the criteria of 15%. The RTD at submaximal torques were calculated evaluating the linear regression between torque and RTD of each limb (see section “Materials and Methods”).

**FIGURE 4:**
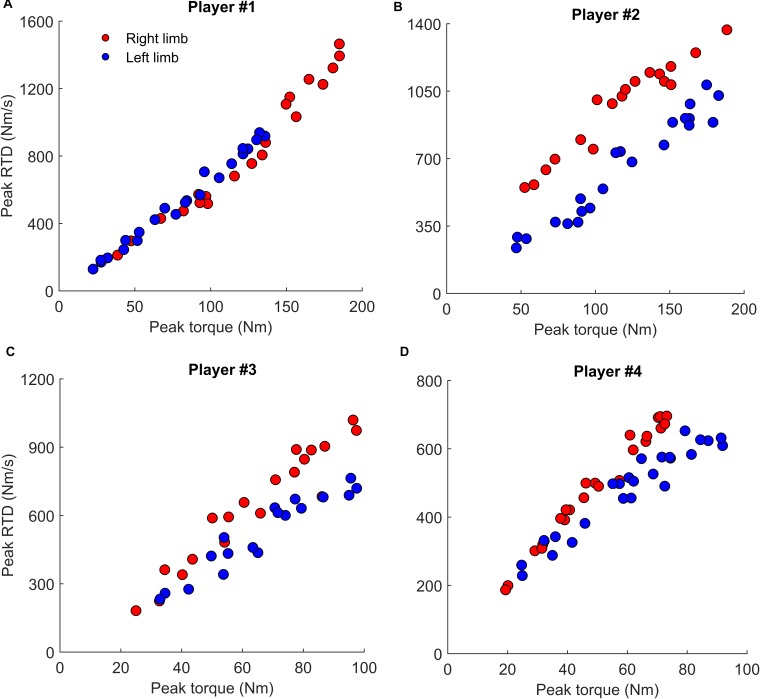
Representative examples of four players performing the isometric protocols consisting in a series of ballistic contractions targeting torque levels from 20 to 100% of maximal torque. Each circle represents the peak torque and rate of torque development (RTD) produced in each ballistic contraction. The right and left limb are reported for descriptive purposes in red and blue, respectively. Panels **(A,B)** are referred to quadriceps muscle, panels **(C,D)** are referred to hamstrings muscle.

At a group level, the range of submaximal torques where the RTD presented an interlimb difference greater than 15%, is presented in Figure 3. For example, a total of 40% (for quadriceps) and 60% (for hamstrings) of players showed an interlimb asymmetry in RTD for at least 50% of torque range. Figure 4 shows the individual values of RTD and peak torque, recorded during the isometric protocol, of four representative players presenting large interindividual differences. Figures 4A,B are related to quadriceps, while Figures 4C,D are related to hamstrings. Extensive comments to the interindividual differences are reported in the discussion sections.

### RTD-SF

The percentages of players not achieving the five different criteria of 5, 10, 15, 20, and 25% of interlimb asymmetry for RTD-SF are reported in Figure 4. Using the cut-off of 15%, ≈70% of players presented an interlimb asymmetry in RTD-SF in quadriceps and/or hamstrings. The average RTD-SF interlimb asymmetry was ≈17% for both quadriceps and hamstrings (Table 1).

**Table 1:** Descriptive statistics of interlimb asymmetries (stronger vs. weaker limb).

		Stronger	Weaker	Difference (90% confidence interval)	Interlimb asymmetry (%)
**Quadriceps**					
	CONC (Nm)	143 ± 25	134 ± 26	7 (6-10)	6.0 ± 4.0
	MVC torque (Nm)	268 ± 55	241 ± 50	27 (17-36)	9.9 ± 8.0
	RTD_max_ (Nm/s)	1557 ± 339	1419 ± 293	138 (94-181)	8.5 ± 6.2
	RTD-SF	7.6 ± 1.6	6.3 ± 1.3	1.3 (1.0-1.7)	17.4 ± 9.1
**Hamstrings**					
	CONC (Nm)	100 ± 20	87 ± 20	13 (8-16)	12.1 ± 9
	MVC torque (Nm)	113 ± 23	102 ± 23	11 (8-13)	10.1 ± 6.2
	RTD_max_ (Nm/s)	882 ± 171	731 ± 184	151 (105-195)	17.1 ± 12.0
	RTD-SF	9.2 ± 1.3	7.6 ± 1.4	1.6 (1.1-2.1)	17.7 ± 10.7


*CONC, peak torque in concentric isokinetic contraction; MVC torque, maximal voluntary isometric contraction torque; RTD_*max*_, maximal rate of torque development; RTD-SF, RTD scaling factor.*

### Comparison Between Indices of Asymmetry

**FIGURE 5:**
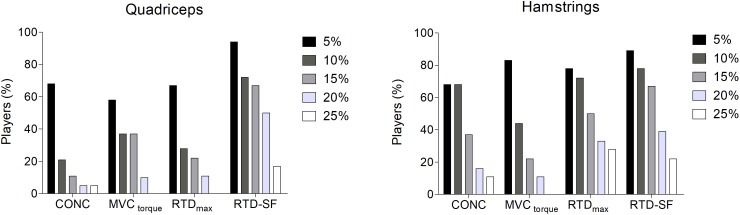
Percentage of players (*y*-axis) not achieving the five different peak torque criteria: 5, 10, 15, 20, and 25% (*x*-axis) for concentric isokinetic contraction torque (CONC), maximal voluntary isometric contraction torque (MVC torque), maximal rate of torque development (RTD_max_), and RTD scaling factor (RTD-SF).

The prevalence of interlimb asymmetry for RTD-SF, RTD_max_, MVC torque, and CONC are presented in Figure 5 for quadriceps and hamstrings. Briefly, regardless the cut-off criteria adopted to identify an asymmetry, the RTD-SF was able to identify more players with asymmetry than any other indices. The average interlimb differences in RTD-SF, RTD_max_, MVC torque, and CONC are reported in Table 1 grouped as stronger vs. weaker limb. Compared to the other indices, the RTD-SF (for both muscle groups) was the index presenting the larger interlimb asymmetry (Table 1).

RTD-SF asymmetry index was found to be moderately correlated with CONC in quadriceps (*r* = 0.61, *p* = 0.009) and with RTD_max_ in hamstrings (*r* = 0.56, *p* = 0.017). All other indices of asymmetry did not correlate with RTD-SF both in quadriceps (MVC torque: *r* = -0.07, *p* = 0.792; RTD_max_: *r* = 0.19, *p* = 0.462) and in hamstrings (MVC torque: *r* = -0.09, *p* = 0.736; CONC: *r* = 0.04, *p* = 0.889).

## Discussion

In this study a sample of young soccer players were tested under isometric and isokinetic conditions to measure interlimb asymmetry in quadriceps and hamstrings. The isometric protocol allowed the evaluation of RTD during ballistic contractions targeting either submaximal or maximal torques. We found that: (1) most players showed and interlimb asymmetry in RTD across a large part of submaximal torques, not only when targeting maximal torque; (2) there was a large prevalence of RTD-SF asymmetry in both quadriceps and hamstrings; (3) the prevalence of asymmetry was larger for RTD-SF than for the other indices of asymmetry, e.g., the isokinetic test.

### Methodological Novelty

The RTD_max_ is usually gathered requesting participants to contract their muscles as fast and as hard as possible. However, while an imbalance in RTD_max_ might be functionally relevant in many occasions, we argue that it would be important to measure possible interlimb asymmetry in ballistic contractions targeting submaximal, rather than maximal, torque levels. The isometric protocol adopted in this study (Bellumori et al., 2011, 2013; Casartelli et al., 2014; Boccia et al., 2018) requested the players to produce a series of ballistic contractions of either submaximal or maximal amplitudes (Figure 2A). This means that the players performed ballistic contractions targeting, as quickly as possible, from ≈20 to ≈100% of MVC torque. The higher was the target torque, the higher was the RTD produced in the contraction (Figures 2B,C). In such a way it was possible to evaluate the RTD across the whole range of torque levels, i.e., when targeting almost all torque levels (Figure 2D). The RTD produced at the end of this protocol, i.e., when asking the participant to reach about the 100% of MVC torque (the upper right values in the Figure 2D) is those usually obtained when assessing the RTD_max_ (Maffiuletti et al., 2016; Rodriguez-Rosell et al., 2017). Conversely, the RTD values obtained when targeting from 20 to 80% of MVC torque are usually overlooked in the literature. Considering that RTD training has been recommended to be incorporated in the sport injury rehabilitation process (Buckthorpe and Roi, 2017), a more comprehensive evaluation of this muscle capacity is warranted.

### RTD in Submaximal Contractions

#### Group Analysis

When analyzing the data at group level, we found that most players showed an interlimb asymmetry (using a cut-off of 15%) in RTD across large part of the range of torque levels (Figure 3). For example, 40% (for quadriceps) and 60% (for hamstrings) of players showed an interlimb asymmetry in RTD for at least 50% of torque range (Figure 3). Hence in these players the interlimb asymmetry in muscle quickness may play a role not only when they try to produce as much torque as possible, but also when targeting submaximal torques.

#### Interindividual Differences

Looking at the Figure 4 it can be possible to understand the valuable information that can be gathered using this protocol and conversely could not be collected if only RTD_max_ is assessed. In the Figure 4, the values of four representative players, which presented large interindividual difference, are reported. The player reported in Figure 4A (quadriceps muscles) behave similarly in the two limbs up to ≈140 Nm. This means that the quickness of the muscle contractions, and the RTD-SF, when targeting values lower than 140 Nm were similar for the two limbs. Nevertheless, the right limb was stronger than the left one, and therefore the former reached higher values of peak torque (≈190 Nm) and RTD. Hence the interlimb asymmetry emerged only at high torque levels and affected both the peak torque and RTD. The player reported in Figure 4B (quadriceps muscles) showed a completely different pattern: while the peak torque reached by the two limbs was similar (≈195 Nm) the RTD, i.e., the quickness of the contraction, was much slower for the left limb than the right one across the whole torque range. This means that the interlimb asymmetry, in terms of muscle quickness, could be seen even in physical tasks requiring very low torque levels. Conversely, the player reported in Figure 4C (hamstrings muscles) showed large interlimb asymmetry in RTD at maximal (≈100 Nm), but not submaximal (≈30 Nm) torque levels. The pattern of the player reported in Figure 4D (hamstrings muscles) is even more complex. While the left limb had higher peak torque and lower RTD, the right limb showed the higher RTD and lower peak torque. Taking together, these representative behaviors allow to understand that the findings that can be gathered when targeting maximal torques, as usually performed, cannot be used to infer what happens when targeting submaximal torques. For this reason, we suggest that the protocol herein adopted may be provide additional meaningful information about the quickness capacity of quadriceps and hamstrings muscles under the whole range of torques.

### Asymmetry in RTD-SF

To identify an interlimb asymmetry as meaningful, various thresholds of difference have been studied, from 5 to 25%, being 10% and 15% the most adopted (Tol et al., 2014). Using the criteria of 15%, ≈70% of players showed an interlimb asymmetry in RTD-SF in both quadriceps and hamstrings. However, the present data did not allow to appreciate if the RTD-SF interlimb asymmetry is functionally relevant. It is established that RTD-SF is an index of muscle quickness that does not depend on maximal torque (Bellumori et al., 2011, 2017), however, the physiological principles underpinning this measure are still to be determined. Even more critical, it is unknown if an asymmetry in RTD-SF can be considered a risk factor for future injuries. However, these experimental questions remain unanswered until further research is conducted. Nevertheless, the fact that we were able to identify many players with an asymmetry makes the RTD-SF a promising index. In line with previous studies (Jones and Bampouras, 2010; Daneshjoo et al., 2013; Coratella et al., 2018), we did not find any appreciable differences in the prevalence of interlimb asymmetry between quadriceps and hamstrings. Finally, the fact that RTD-SF interlimb difference in hamstrings was related to leg dominance, makes the study of this variable even more potentially interesting.

### Comparison Between Indices of Asymmetry

Regardless the threshold adopted to define an asymmetry as meaningful (Tol et al., 2014), the RTD-SF was able to identify more players with asymmetry than any other indices (Figure 5). For example, using the cut-off of 15%, the RTD-SF identified ≈70% of players with an interlimb asymmetry in hamstrings. Conversely, the concentric isokinetic test identified only ≈40% in the same muscle. Furthermore, the average value of RTD-SF interlimb asymmetry, expressed as weaker vs. stronger limb, was greater than the other indices (Table 1). For example, while the average RTD-SF asymmetry was about ≈17% in both quadriceps and hamstrings, the peak torque in concentric contraction presented an interlimb difference of ≈6% in quadriceps and ≈12% in hamstrings (Table 1).

With few exception, RTD-SF asymmetry index did not correlate with the other interlimb indices calculated herein. This result confirms previous researches founding poor or no correlations between various indices of asymmetry (Thomas et al., 2017). For example, a recent study found that the hamstrings peak torque measured under isokinetic condition and Nordic hamstrings exercise test were poorly correlated (van Dyk et al., 2018). These findings are expected since each test is biomechanically different in nature with respect to the others. However, RTD-SF asymmetry index moderately correlated with peak torque in concentric contraction in quadriceps and RTD_max_ in hamstrings. These correlations are difficult to interpret though. Indeed, it is difficult to explain why, for example, the RTD-SF should be related to the peak torque in concentric contraction in quadriceps but not in hamstrings. It is more likely, that the moderate correlations that we found resulted statistically significant just for chance, rather than a physiological connection between the measurements.

### Limitations of the Study

The main limitation of this study was the small sample size, which does not allow to generalize the present findings to the populations of soccer players. Moreover, since all players were younger than 19 years of age, the findings of this study may have been affected by biological maturations of the participants and thus should not be applied to adult players. Furthermore, since we adopted single-joint exercises to test muscle strength, nothing can be said about what could happen when adopting more functional multi-joint exercises. Finally, we did not test if the asymmetries highlighted in this study were related to on-court performance and/or can be considered as risk factors for future injuries.

## Conclusion and Future Perspectives

Evaluating the RTD in ballistic contraction targeting submaximal maximal torques is usually overlooked in the literature. However, it provided meaningful information that could not be gathered when adopting the classical method of ballistic contractions targeting maximal torques. We reported that most players presented an interlimb asymmetry in RTD for a wide part of the torque range and that the adopted protocol was able to highlight important interindividual differences. Furthermore, the herein adopted protocol allowed to calculate the so-called RTD scaling factor, which was able to identify even more players with interlimb asymmetry than the widely adopted method of isokinetic tests. However, if these asymmetries are functionally relevant and can be considered as risk factors for future injuries is still to be determined.

## Author Contributions

GBo conceived the study. GBo, PRB, RC, and AR contributed methodology. GBo, PRB, GBu, MC, MB, RC, and AR investigated the study, and reviewed and edited the manuscript. GBo and PRB performed the formal analysis and drafted the original manuscript. RC and AR supervised the study.

## Conflict of Interest Statement

MB was employed by company Atalanta BC. The remaining authors declare that the research was conducted in the absence of any commercial or financial relationships that could be construed as a potential conflict of interest.
